# Endothelial cells release soluble factors that support the long-term survival of filarial worms *in vitro*

**DOI:** 10.1016/j.exppara.2016.08.004

**Published:** 2016-11

**Authors:** Holly Evans, Alexander Francis Flynn, Edward Mitre

**Affiliations:** Department of Microbiology and Immunology, Uniformed Services University of the Health Sciences, 4301 Jones Bridge Road, Bethesda, MD 20814, United States

**Keywords:** Filariae, *In vitro* cell culture, Endothelial cells, Life cycle

## Abstract

The inability to maintain filarial nematodes in long-term *in vitro* culture greatly limits research into the basic biology of these parasites and hinders *in vitro* screening of novel anti-filarial agents. In this study, we sought to characterize nutrients that promote the long-term survival of filarial worms *in vitro*. Using microfilariae (MF) obtained from gerbils infected with *Litomosoides sigmodontis*, a filarial parasite of rodents, we found that Dulbecco's Modified Eagle Medium (DMEM) supplemented with 10% fetal bovine serum (FBS) resulted in MF survival of only 5 days. However, co-culturing MF with a mouse endothelial cell line (EOMA) enabled survival for 40 days. Culturing EOMA cells in transwell plates extended MF survival to the same degree as direct co-culture, suggesting that the factors microfilariae require are soluble in nature. Heat inactivation of EOMA conditioned media at 56 °C reduced MF survival by approximately 50%, and heat inactivation at 100 °C reduced survival to 3 days, demonstrating that both heat labile and heat stable factors are involved. EOMA cells require FBS to produce these factors, as conditioned media collected from EOMA cells grown in the absence of FBS failed to prolong survival. The removal of lipids also abrogated survival, indicating MF are likely utilizing lipid factors released by EOMA cells. Dialysis experiments demonstrate that at least some of the required factors are between 0.1 and 1 kDa in size. Importantly, *L. sigmodontis* adult worms also show significantly extended survival when cultured in EOMA conditioned media. Together, these results suggest that EOMA-produced factors include lipid-containing molecules, heat labile molecules (likely a protein), and micronutrients between 0.1 and 1 kDa in size. These studies have established a cell-free approach to maintaining MF and adult stage filarial worms in long-term *in vitro* culture and have taken important steps towards biochemically characterizing host-derived nutrients required for parasite survival.

## 1 Introduction

Filariae are tissue-invasive roundworms transmitted by arthropods. Pathogenic human filariae include *Wuchereria bancrofti* and *Brugia malayi*, which cause lymphatic filariasis (elephantiasis), and *Onchocerca volvulus*, the agent of river blindness. These diseases cause tremendous morbidity worldwide. A major factor limiting the effectiveness of current mass drug administration (MDA) efforts to control, and potentially eradicate lymphatic filariasis and onchocerciasis, is the inability of current anti-filarial drugs to kill adult worms, especially when given as single dose treatments. Because these medicines kill microfilariae (MF) but not adult worms, MDA has to be given repeatedly for several years until natural death of adult worms occurs.

Compared to other infectious organisms, little is known about the basic biology of filarial worms. Understanding the specific nutrients filarial worms require to survive, reproduce, and molt provides a foundation for rational drug development. Specific drugs can be designed to target nutrient uptake, sexual differentiation, reproduction, MF development and release, molting, motility, and migration. Drugs that affect adult worm longevity would be particularly important for the Global Program to Eliminate Lymphatic Filariasis, as incorporation of macrofilaricidal drugs into the program would drastically shorten the time required for the elimination of filarial worms in endemic regions. However, obstacles to development of novel antibiotics against adult filarial worms include: 1) inability to maintain filarial worms for long periods of time *in vitro* in cell-free culture, 2) limited understanding of the host factors required for parasite survival, and 3) need to obtain filarial worms from infected animals.

An *in vitro* culture system that enables worm longevity would greatly enhance screening and discovery of novel drug candidates and would facilitate our ability to study the basic biology of these organisms. While there have been previous efforts to optimize the *in vitro* culture of filarial worms, there have been no substantive efforts in this direction for over fifteen years. To date, the majority of *in vitro* culture work has focused on worm molting (Nelson et al., 1982, Abraham et al., 1990, Lok et al., 1984, Mak et al., 1983, Ramesh et al., 2005). Very little is known about the basic nutrients required to support *in vitro* survival and to date there has not been a cell-free method of maintaining filariae in long-term culture.

There is evidence that co-culturing filarial worms with cell lines enhances worm survival and molting (Smyth, 1990). While others have shown that feeder cell lines are important (Townson et al., 1986, Townson et al., 2006), no one has characterized or identified the molecules necessary for survival. We found two cell lines that substantially prolong *in vitro* culture of both MF and adult filarial worms, demonstrated that the released factors are soluble, and conducted a series of biochemical tests that characterized these beneficial factors. Results from this study provide a method using conditioned media to maintain filariae *in vitro*. Additionally, this work represents the first steps toward the development of a chemically defined medium that provides filarial worms the nutrients required for long-term *in vitro* survival.

## 2 Methods

### 2.1 Microfilariae isolation and culture

We chose to use *Litomosoides sigmodontis,* a filarial parasite of rodents, because it is an excellent filarial species for testing antifilarial agents. It was the model used to discover diethylcarbamazine by Lederle labs in the 1940s (Gupta, 1987), and it has been shown to exhibit similar susceptibility profiles to antifilarial agents as *Brugia malayi* (Zhner and Schares, 1993). Microfilariae (MF) were isolated from the blood of *Litomosoides sigmodontis*-infected jirds (*Meriones unguiculatus)* obtained from TRS Laboratory (Athens, GA). A terminal bleed was performed and pooled blood from 2 to 3 infected jirds was collected into a heparinized microcentrifuge tube (BD) and added to 2 ml of RPMI-1640 (Mediatech, Inc.). MF were then isolated from the blood via percoll gradient centrifugation as previously described (Chandrashekar et al., 1984). In brief, a 25% Percoll solution was laid on top of a 30% Percoll solution in a 15 ml conical. The blood sample was then carefully layered above the Percoll solutions and centrifuged at 400 × g for 35 min with no brake. The layer containing MF was transferred to a new tube, washed with RPMI, and centrifuged at 400 × g for 10 min. Supernatants were discarded and MF resuspended in 1–2 ml RPMI. MF were then counted on a hemacytometer. For all experiments, MF were cultured in triplicate at a concentration of 2 × 10^4^ MF/ml. *In vitro* cultures of microfilariae were maintained in the absence of media changes. Viability was determined on the basis of motility, which was evaluated by microscopy on a daily basis.

### 2.2 Cell lines and culture conditions

A murine endothelial cell line (EOMA) (CRL-2586), a murine myeloma cell line (Sp2/0-Ag14, CRL-1581), a mosquito cell line (C6/36) (CRL-1660), and rat basophil-like cell line (RBL-2H3) (CRL-2256) were obtained from the American Type Culture Collection (ATCC). EOMA and myeloma cells were maintained in Dulbecco's Modified Eagles Medium (DMEM) and RBL-2H3 cells were maintained in Iscove's Modification of DMEM (IMDM) at 37 °C and 5% CO_2_. C6/36 cells were maintained in Eagles Minimum Essential Medium (EMEM) and cultured at 28 °C and 5% CO_2_. Unless otherwise noted, all cell culture media were supplemented with 10% FBS, 2 mM L-glutamine, and 100 U/ml penicillin/streptomycin. Cell culture reagents were purchased from MediaTech, Inc.

### 2.3 Transwell plates

For transwell plate culture, EOMA cells in 2 ml of supplemented DMEM were added to the bottom reservoir of 0.4 μm transwell plates (Corning, Inc.). MF in 2 ml of supplemented DMEM were then added to the top reservoir.

### 2.4 Conditioned media

Cells were passaged into 182 cm^2^ vented tissue culture flasks (Cell Treat). 24 h after passage, media was removed and 50 ml fresh media was added. Three days later, the conditioned media (CM) was harvested and centrifuged at 400 × g for 10 min. The supernatant was collected, pooled, and sterile filtered. CM was flash frozen in a dry ice and ethanol slurry and aliquots were stored at −80 °C for future use. For some experiments, EOMA CM was prepared in the absence of FBS by growing cells for three days in DMEM supplemented with 2 mM L-glutamine and 100 U/ml penicillin/streptomycin.

### 2.5 Heat treatment

Samples were placed in a dry heat bath set at 56 °C or 100 °C. Samples were incubated for 1 or 4 h, cooled to room temperature, and then supplemented with 100 U/ml of penicillin/streptomycin. For some experiments, 10% FBS was added after heat treatment.

### 2.6 Lipid depletion

Lipid removal agent (LRA) (Advanced Minerals) was added to 10 ml EOMA CM and DMEM for a final concentration of 40 mg/ml. Samples were agitated at room temperature for 22 h and then centrifuged at 4696 × g for 10 min. Supernatants were removed and centrifuged again at 4696 × g for 10 min. Supernatants were then passed through a 0.22μm filter, flash frozen, and stored at −80 °C.

### 2.7 Dialysis

5 ml of EOMA CM was added to 1000 kDa, 20 kDa, 1 kDa, and 0.1 kDa Float-A-Lyzer G2 dialysis devices (Spectrum Laboratories, Inc.) pre-conditioned as per manufacturer's protocol. Dialysis devices were placed in 600 ml dialysis buffer (DMEM supplemented with 10% FBS, 2 mM L-glutamine, and 100 U/ml penicillin/streptomycin) with a stir bar on a magnetic plate at room temperature. Buffer was exchanged at 2, 4, 6, and 18 h after the last buffer exchange, CM was harvested, flash frozen, and stored at −80 °C.

### 2.8 2D gel

Aliquots of frozen EOMA CM and RBL-2H3 CM were sent to Applied Biomics (Hayward, CA) for 2D DIGE protein expression profiling. Spots that showed greater than 1.5 fold up-regulation in EOMA CM compared to RBL-2H3 CM were identified by mass spectrometry. A cluster analysis was then performed on the identified proteins to determine which functional groups (GO terms) were enriched in EOMA CM.

### 2.9 Culture supplements

In place of FBS, 10% Cell-*Ess*^®^ (Essential Pharma) or 1X Fatty Acid Supplement (Sigma) was added to DMEM with 2 mM L-glutamine and 100 U/ml penicillin/streptomycin. To supply exogenous purines, EmbryoMax Nucleosides (Millipore) was added at a 1X concentration to DMEM with 10% FBS, L-glutamine and penicillin/streptomycin. Finally, 10 μg/ml cholesterol (Sigma), 20 μg/ml yeast-derived recombinant mouse C3 (MyBiosource), 20 μg/ml yeast-derived recombinant apolipoprotein E (MyBiosource), and 20 μg/ml native albumin (MyBiosourse) were added individually to DMEM with 10% FBS, L-glutamine, and penicillin/streptomycin.

### 2.10 Adult worm culture

Adult *L. sigmodontis* worms were isolated from the pleural cavity of infected jirds (*Meriones unguiculatus)* obtained from TRS Laboratory (Athens, GA). For transwell plate experiments, EOMA cells in 2 ml of supplemented DMEM was added to the bottom reservoir of 0.4 μm transwell plates, and 1 adult female in 2 ml of supplemented DMEM was added to the top reservoir. For conditioned media experiments, 1 adult female worm was cultured in 1 ml CM and media was exchanged every other day.

### 2.11 Statistical analysis

For all experiments, MF or adult worms were cultured in triplicate for each condition tested. All experiments were repeated except for cell co-culture and transwell experiments. Points on graph and statistical analyses used combined data from all experimental replicates. To estimate median survival time for MF, a 3- or 4-parameter logistic curve was used (depending on which form fit the data best) with a variable slope and top and bottom parameters constrained to 100 and 0, respectively. Median survival times were compared pairwise between experimental groups using an F test and the Bonferroni correction for multiple comparisons. Survival curves for adult *L. sigmodontis* worms were compared between DMEM and EOMA CM using a log-rank (Mantel-Cox) test.

### 2.12 Ethics statement

All experiments were performed under protocol #12-173 “Establishment and maintenance of *Litomosoides sigmodontis* lifecycle in jirds (*Meriones unguiculatus*)” approved by the Uniformed Services University Institutional Animal Care and Use Committee. This protocol adheres to the USDA Animal Welfare Regulations PHS Policy on Humane Care and Use of Laboratory Animals published by the National Research Council (8th Edition, 2011).

## 3 Results

### 3.1 Co-culturing MF with EOMA cells extends *in vitro* survival from 5 days to 40 days

**Fig. 1:**
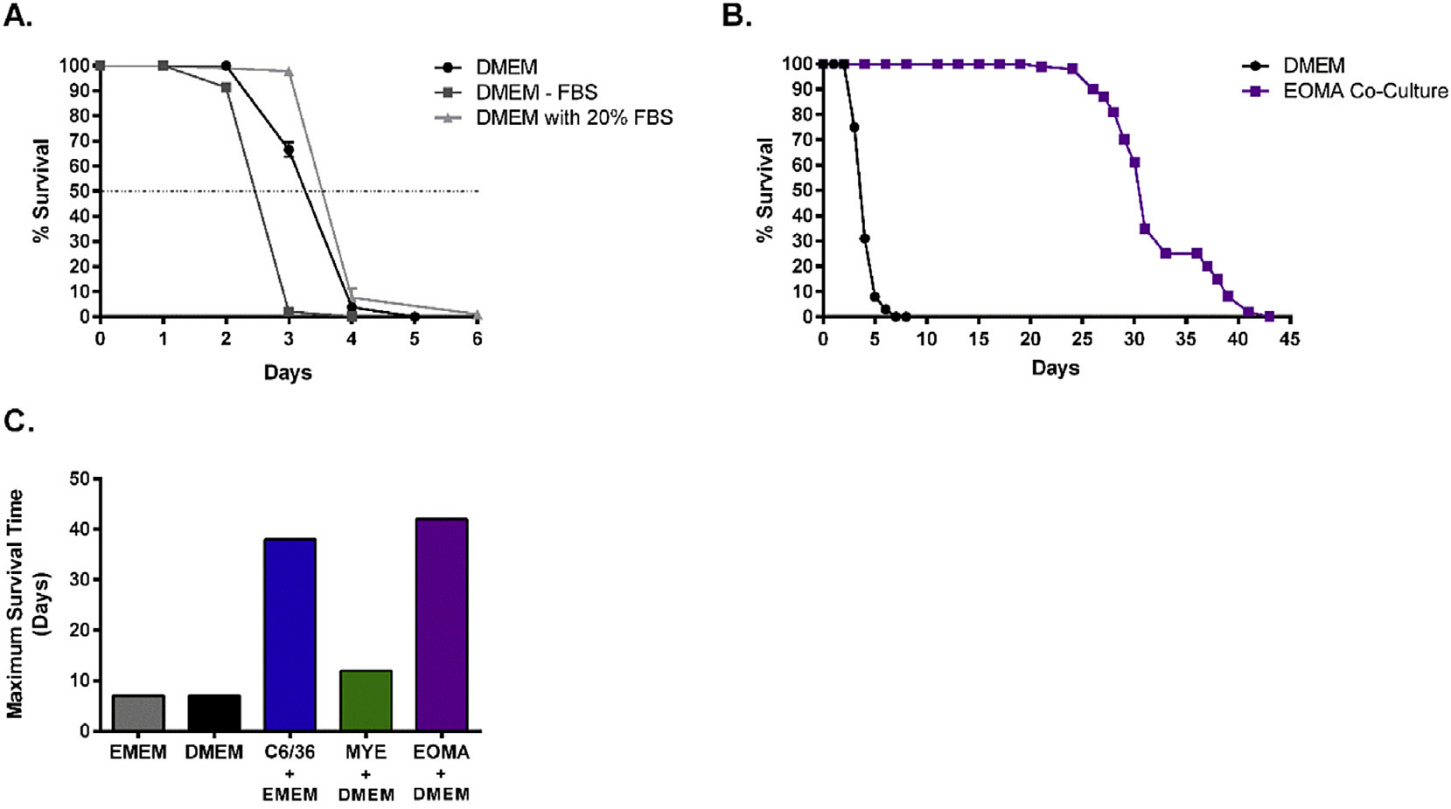
Co-culture with mouse endothelial cell and mosquito lines enhances *in vitro* survival of MF. (A) MF survival in DMEM supplemented with various concentrations of FBS. DMEM = DMEM with 10% FBS. DMEM – FBS = DMEM with 0% FBS. DMEM 20% FBS = DMEM with 20% FBS. All groups were significantly different from one another. Median survival was calculated as 2.38 days for DMEM – FBS, 3.17 days for DMEM, and 3.61 days for DMEM + 20% FBS. (B) Co-culture of MF with a murine endothelial cell line in DMEM (EOMA co-culture) or with DMEM alone. (C) Maximum duration of MF survival when co-cultured with mosquito (C6/36), murine myeloma (MYE), and EOMA cell lines. As a control, MF were cultured in EMEM and DMEM supplemented with 10% FBS.

To characterize MF survival in basic cell culture media, we cultured MF in Dulbecco's Modified Eagles Medium (DMEM) supplemented with 10% FBS (Fig. 1A). We found that MF survive for a maximum of 5 days in this medium. Raising the FBS concentration to 20% did not greatly extend survival, however lowering FBS to 0% expedited worm death. Although statistically significant, differences in median survival times varied by less than one day. Unless otherwise noted, all subsequent experiments were performed with DMEM supplemented with 10% FBS.

We first chose to co-culture MF with a mouse endothelial cell line (EOMA) because MF circulate within the blood stream of the mammalian host. When MF were co-cultured with EOMA cells grown in DMEM, survival was greatly enhanced (Fig. 1B). MF were able to survive for up to 40 days with no media exchange. During the life cycle, circulating MF are ingested by a vector during a bloodmeal. To test whether cells from an insect vector could also support survival, MF co-culture with a mosquito cell line (C6/36). Survival was indeed enhanced when MF were co-cultured with C6/36 cells grown in Eagles Minimum Essential Medium (EMEM) supplemented with 10% FBS. Not all cell lines were capable of supporting MF survival, as MF cultured with a mouse myeloma cell line only lived for 10 days (Fig. 1C).

### 3.2 EOMA cells release soluble factors that are constitutively produced when cells are grown in the presence of FBS

**Fig. 2:**
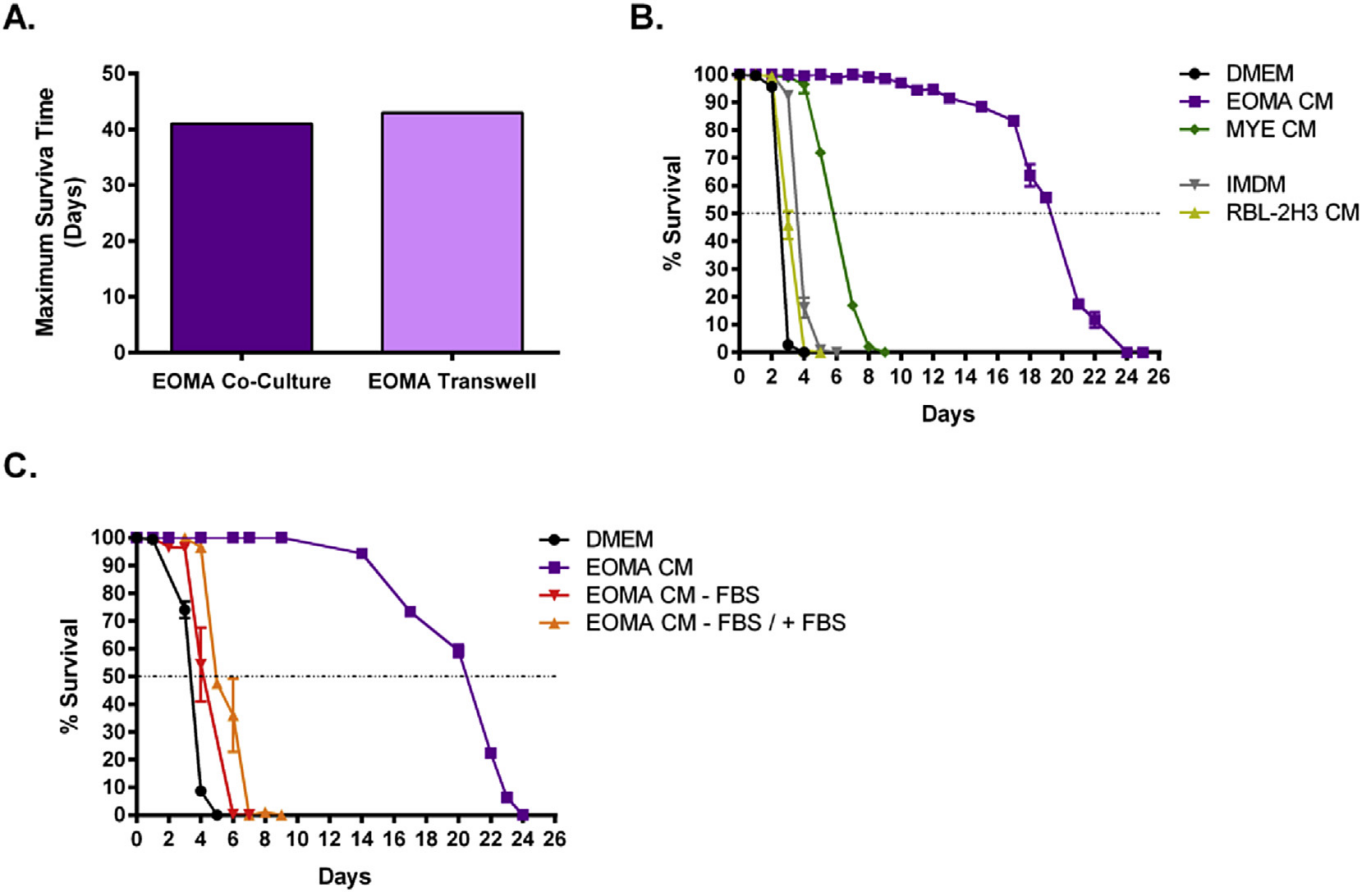
Factors that prolong survival are soluble and constitutively produced when EOMA cells are grown in the presence of FBS. (A) Maximum duration of MF survival when cultured with EOMA cells in 0.22 μm transwell plates. (B) MF survival in CM from various cell lines. All groups were significantly different from one another, except for DMEM vs. RBL-2H3 CM. Median survival was calculated as 2.46 days for DMEM, 2.97 days for RBL CM, 3.60 for IMDM, 5.76 days for MYE CM, and 19.01 days for EOMA CM. (C) MF survival in CM derived from EOMA cells grown for 3 days in the absence of FBS. DMEM = DMEM with 10% FBS. EOMA CM = CM from EOMA cells grown in DMEM with 10% FBS. EOMA CM − FBS = CM from EOMA cells grown in DMEM without FBS. EOMA CM – FBS/+FBS = CM of EOMA cells grown in DMEM without FBS, followed by subsequent addition of 10% FBS. All groups were significantly different from one another. Median survival was calculated as 3.31 days for DMEM, 4.06 days for EOMA CM − FBS, 5.41 days for EOMA CM − FBS/+FBS, and 19.84 days for EOMA CM.

To test whether MF require soluble factors released by EOMA cells or direct contact with the cells to achieve prolonged survival, we cultured MF and EOMA cells in transwell plates. EOMA cells were cultured in the bottom reservoir and MF were cultured in the top reservoir, with the two reservoirs separated by a 0.22 μm filter. Culturing MF in transwell plates with EOMA cells resulted in similar survival rates as co-culture, indicating that prolonged survival was due to soluble factors (Fig. 2A).

All stages of filarial worms release excretory-secretory products that have been shown to have diverse effects on host cells. We next sought to determine whether MF were inducing EOMA cells to produce survival factors, or if the cells were producing these factors constitutively. EOMA cells were cultured for 3 days in the absence of MF and the CM was collected. When MF were cultured in CM survival was also greatly enhanced, with a median survival of 19 days (Fig. 2B). This indicates that EOMA cells constitutively produce factors that MF require for survival. Culturing MF in myeloma CM did not produce robust results, with median survival of ∼6 days. Finally, culturing MF in CM from a rat basophilic leukemia cell line (RBL-2H3) did not extend survival beyond the media control (IMDM with 10% FBS).

FBS is a complex mixture of nutrients and can exhibit a high degree of batch to batch variability. In an effort to simplify culture conditions so that we may characterize the factors MF need to survive, we cultured EOMA cells in the absence of FBS (Fig. 2C). Interestingly, enhanced survival is not observed when MF are cultured in CM from cells grown in the absence of FBS (EOMA CM − FBS). Furthermore, subsequent addition of 10% FBS to EOMA CM − FBS (EOMA CM − FBS/+FBS) did not restore MF survival. This indicates that EOMA cells require FBS to produce the factors that are beneficial for MF survival.

### 3.3 Factors that support MF survival are sensitive to heat treatment and lipid depletion, and are between 0.1 and 1 kDa in size.

**Fig. 3:**
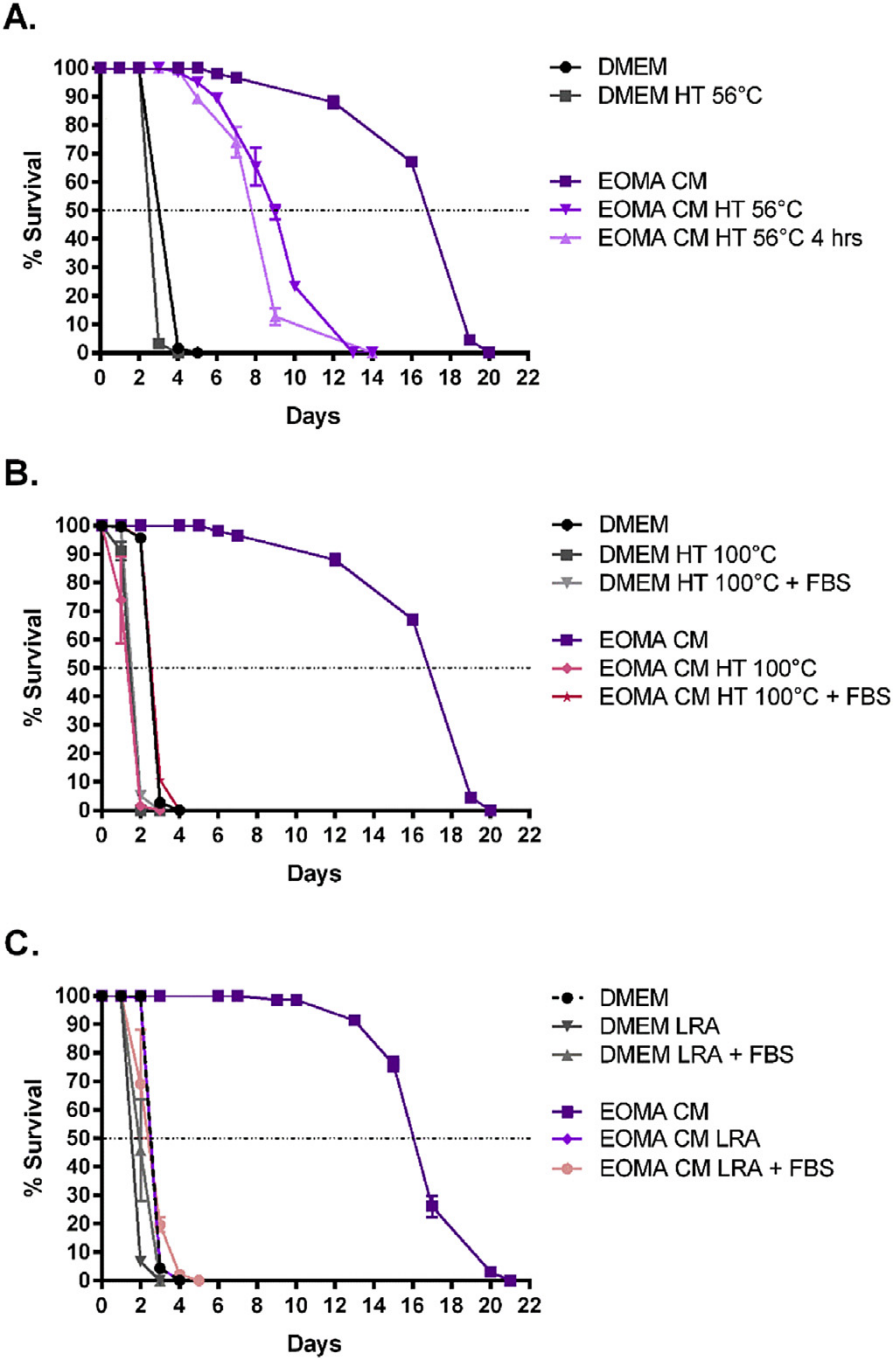
EOMA CM contains factors that are sensitive to heat treatment and lipid depletion. (A) MF survival in DMEM and EOMA CM heat treated at 56 °C for 1 or 4 h. All groups were significantly different from one another, except for DMEM vs. DMEM HT 56 °C. Median survival was calculated as 2.88 days for DMEM HT 56 °C, 3.6 days for DMEM, 7.69 days for EOMA CM HT 56 °C 4 h, 8.82 days for EOMA CM HT 56 °C, and 16.57 days for EOMA CM. (B) MF survival in DMEM and EOMA CM heat treated at 100 °C for 1 h. DMEM HT 100 °C + FBS = DMEM heat treated at 100 °C for 1 h followed by subsequent addition of 10% FBS. EOMA CM HT 100 °C + FBS = EOMA CM heat treated at 100 °C for 1 h followed by subsequent addition of 10% FBS. Groups were statistically different from one another except for: DMEM vs. EOMA CM HT 100 °C + FBS, DMEM HT 100 °C vs. DMEM HT 100 °C + FBS, DMEM HT 100 °C vs. EOMA CM HT 100 °C, and DMEM HT 100 °C + FBS vs. EOMA CM HT 100 °C. Median survival was calculated as 1.34 days for EOMA CM HT 100 °C, 1.53 days for DMEM HT 100 °C, 1.54 days for DMEM HT 100 °C + FBS, 2.49 days for DMEM, 2.55 days for EOMA CM 100 °C, and 16.31 days for EOMA CM. (C) MF survival in DMEM and EOMA CM depleted of lipids and lipoproteins with LRA. DMEM LRA = DMEM treated with LRA. DMEM LRA + 10% FBS = DMEM treated with LRA, followed by subsequent addition of 10% FBS. EOMA LRA = EOMA CM treated with LRA. EOMA LRA + 10% FBS = EOMA CM treated with LRA, followed by subsequent addition of 10% FBS. EOMA CM was statistically different from all other groups. Additionally, DMEM vs. DMEM LRA, DMEM LRA vs. EOMA CM LRA, and DMEM LRA vs. EOMA CM LRA + FBS were statistically different from one another. Median survival was calculated as 1.90 days for DMEM LRA, 1.99 days for DMEM LRA + FBS, 2.36 days for EOMA CM LRA + FBS, 2.62 days for EOMA CM LRA, 2.88 days for DMEM, and 16.03 days for EOMA CM.

To elucidate the biochemical properties of the factors supporting MF survival, we performed heat treatment, lipid depletion, and dialysis on EOMA CM. Heat treating EOMA CM at 56 °C for 1 h resulted in a 50% reduction in worm survival (Fig. 3A). Heat treating the CM for a longer period of time (4 h) did not further reduce survival. Importantly, MF survival was not significantly affected by heat treating DMEM at 56 °C (median survival of 2.88 days) compared to DMEM (median survival of 3.6 days). This indicates that EOMA CM contains heat labile factors which may potentially be proteins.

Heat treatment of EOMA CM at 100 °C for 1 h eliminated the enhanced survival conferred by EOMA CM (Fig. 3B). The addition of 10% FBS to EOMA CM after 100 °C heat treatment did not reverse the effects of 100 °C heat treatment. MF survival in 100 °C heat treated DMEM fell below the unheated DMEM control, regardless of whether or not 10% FBS was added after heat treatment. These data indicate that EOMA CM also contains heat stable factors that MF require for survival.

We next used a commercially available lipid removal agent (LRA) to deplete EOMA CM of lipids and lipoproteins (Fig. 3C). Longevity of MF in lipid-depleted EOMA CM was similar to the untreated DMEM control. This suggests the worms require a lipid factor released by EOMA cells. Because FBS contains lipids, we supplemented EOMA CM with 10% FBS after the LRA treatment, however it did not appreciably enhance survival. LRA treatment of DMEM resulted in similar median survival time (1.90 days) as DMEM control media that did not undergo lipid removal (2.88 days), as well as that of LRA-treated EOMA CM (2.36 days).

**Fig. 4:**
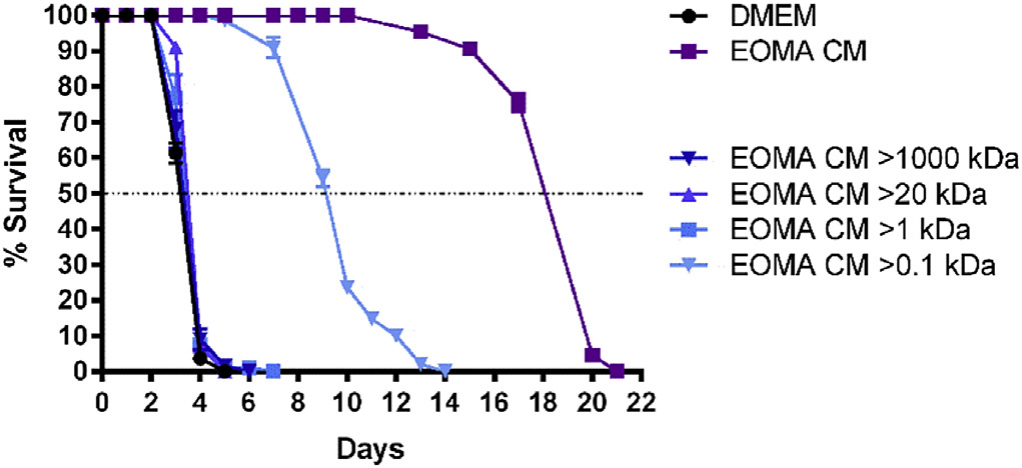
**MF require factors between 0.1**–**1 kDa in size**. MF were cultured in EOMA CM dialyzed into DMEM using 1000, 20, 1, and 0.1 kDa molecular weight cutoffs. DMEM = DMEM supplemented with 10% FBS. EOMA CM > 1000 kDa = EOMA CM including all factors greater than 1000 kDA. EOMA CM > 20 kDa = EOMA CM including all factors greater than 20 kDa. EOMA CM > 1 kDa = EOMA CM including all factors greater than 1 kDA. EOMA CM > 0.1 kDA = EOMA CM including all factors greater than 0.1 kDa. All groups were statistically different from one another except for: DMEM vs. EOMA CM > 1000 kDa, and EOMA CM > 1000 kDa vs. EOMA CM > 1 kDa. Median survival was calculated as 3.12 days for DMEM, 3.25 days for EOMA CM > 1000 kDa, 3.32 days for EOMA CM > 1 kDa, 3.48 days for EOMA CM > 20 kDa, 9.10 days for EOMA CM > 0.1 kDa, and 17.83 days for EOMA CM.

We next performed dialysis to determine the size of the factors responsible for enhanced MF survival. EOMA CM was dialyzed into DMEM using several molecular weight cutoffs (Fig. 4). The only fraction that resulted in enhanced MF survival was the 0.1 kDa cutoff, which contains all molecules in EOMA CM except those less than 0.1 kDa in size. Interestingly, the 1 kDa cutoff did not show enhanced survival. This fraction consists of EOMA CM lacking factors smaller than 1 kDa. Together, these results suggest that the size of at least some of the beneficial factors falls between 0.1 and 1 kDa. Since none of the fractions completely restored MF survival to that of the EOMA CM control, it is likely that a combination of large and small factors are acting in concert to promote MF longevity.

### 3.4 C3 is up-regulated in EOMA CM and enhances MF survival

**Fig. 5:**
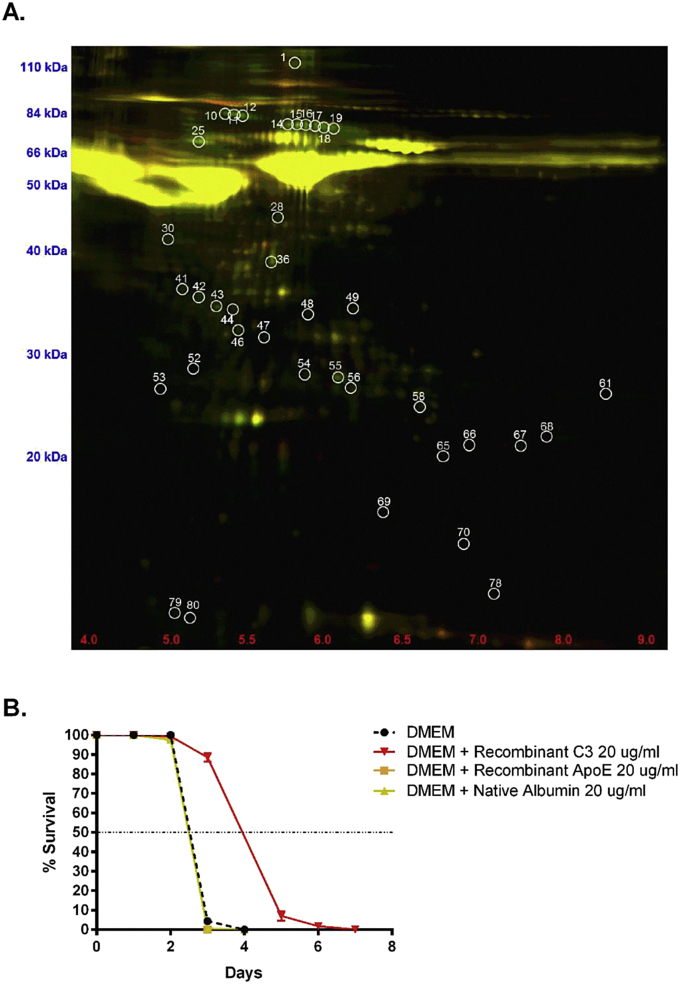
C3 is upregulated in EOMA CM and modestly prolongs MF survival. (A) Comparative 2D DIGE gel of EOMA CM (green) and RBL-2H3 CM (red). 32 spots that exhibited >1.5 fold upregulation in EOMA CM were analyzed by mass spectrometry (Table 1). A cluster analysis was performed to determine which functional groups were enhanced in EOMA CM (Table 2). (B) Supplementation of DMEM with proteins from Table 2 that were categorized as secreted/extracellular. A single dose of 20 μg of recombinant mouse C3, recombinant apolipoprotein E, or native albumin was added to DMEM with 10% FBS. DMEM + Recombinant C3 was statistically different from all other groups. Median survival was calculated as 2.15 days for DMEM + Recombinant ApoE, 2.41 days for DMEM + Native Albumin, 2.88 days for DMEM, and 3.89 days for DMEM + Recombinant C3.

Heat treatment of EOMA CM at 56 °C reduced MF survival, suggesting that MF may require proteins present in EOMA CM. We ran a comparative 2D gel to determine which proteins are up-regulated in EOMA CM compared to RBL-2H3 CM, which does not support MF survival. There were 32 unique spots that were up-regulated in EOMA CM (Fig. 5A), which we then identified by mass spectrometry (Table 1). A cluster analysis of the up-regulated proteins resulted in four functional groups: regulation of cell death, secreted/extracellular, nucleotide binding, and metal ion binding (Table 2).

Due to the soluble nature of the factors that support MF survival, we were interested in supplementing DMEM with candidate proteins from the secreted/extracellular functional group. These included albumin, apolipoprotein E, C3, and collagen. The addition of a single dose of 20 μg of recombinant mouse C3 slightly extended the *in vitro* survival of MF, whereas recombinant apolipoprotein E and purified albumin did not prolong survival (Fig. 5B). The addition of purified collagen was toxic to the MF (data not shown), potentially due to the buffer in which collagen was stored.

### 3.5 Lipid and nucleoside supplements do not prolong MF survival

**Fig. 6:**
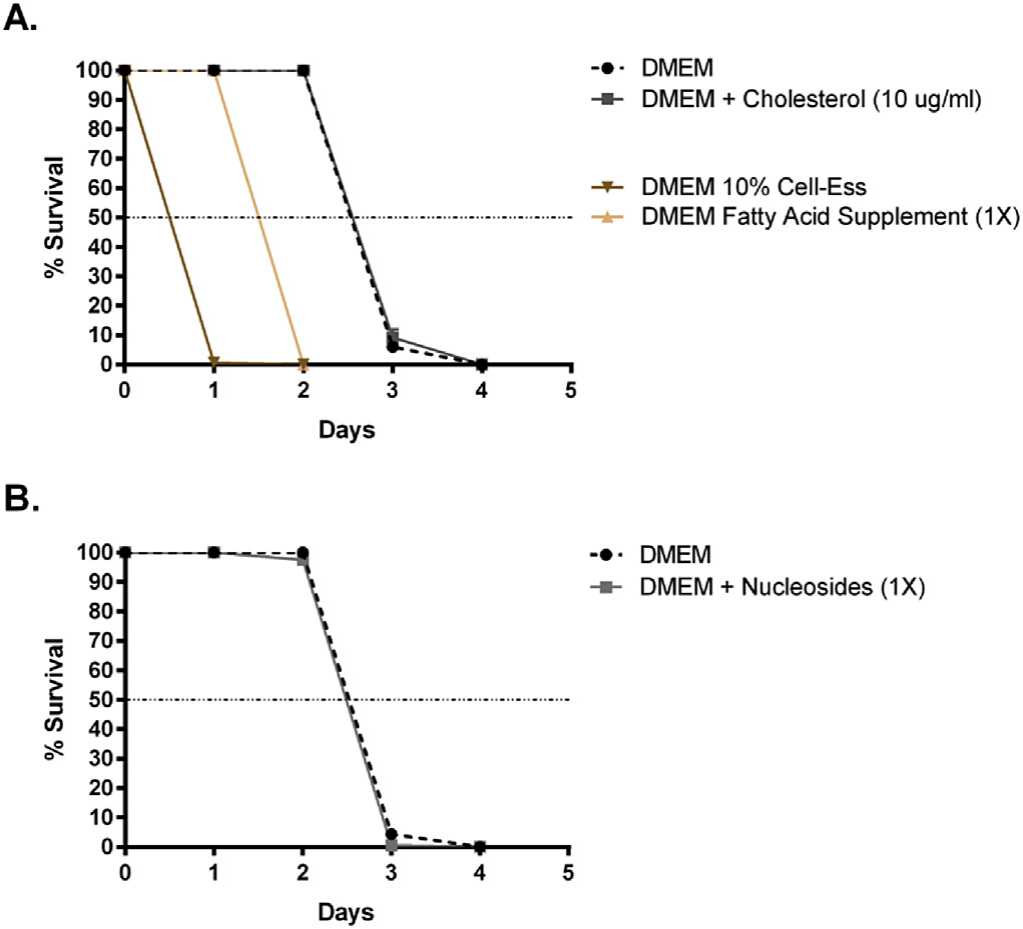
The use of serum replacements, added cholesterol, or nucleosides did not enhance MF survival. (A) 10% Cell-Ess or 1X Fatty Acid Supplement was added to unsupplemented DMEM as serum replacements. Additionally, 10 μg/ml of cholesterol was added to DMEM with 10% FBS. All groups were statistically different from one another except for DMEM vs. DMEM + Cholesterol. Median survival was calculated as 0.05 days for DMEM 10% Cell-Ess, 1.50 days for DMEM + Fatty Acid Supplement, 2.54 days for DMEM, and 2.57 days for DMEM + Cholesterol. (B) A nucleoside supplement (1X) was added to DMEM with 10% FBS. There was no difference between groups. Median survival was calculated as 2.49 days for DMEM + Nucleosides, and 2.53 days for DMEM.

In previous studies, mammalian-derived serum was the only lipid-rich cell culture supplement available for culturing filarial worms *in vitro* (Smyth, 1990). With chemically defined serum replacements now commercially available, we sought to determine whether they could support MF survival. Cell-Ess^®^ is a synthetic serum replacement that contains lipids and growth factors for *in vitro* cell culture. Despite supporting the growth of primary cells and cell lines, the addition of 10% Cell-Ess^®^ to unsupplemented DMEM was toxic to MF (Fig. 6A). Similarly, when a Fatty Acid Supplement (Sigma) was added to unsupplemented DMEM, MF had a shorter median survival time (1.50 days) compared to DMEM (2.54 days) (Fig. 6A).

Filarial worms have previously been shown to lack pathways for *de novo* synthesis of sterols (Furlong, 1989). Although FBS contains cholesterol, we wanted to determine if the addition of extra cholesterol would be beneficial for the MF. We added 10 μg/ml of cholesterol to DMEM, but found it had no effect on MF survival (Fig. 6A). Finally, because filarial worms also lack pathways for *de novo* purine synthesis, and because nucleotide binding proteins were a functional group that was up-regulated in EOMA CM, we added a nucleoside supplement to DMEM. However the addition of exogenous nucleosides had no effect on worm survival (Fig. 6B).

### 3.6 EOMA CM prolongs survival of adult *L. sigmodontis* worms

**Fig. 7:**
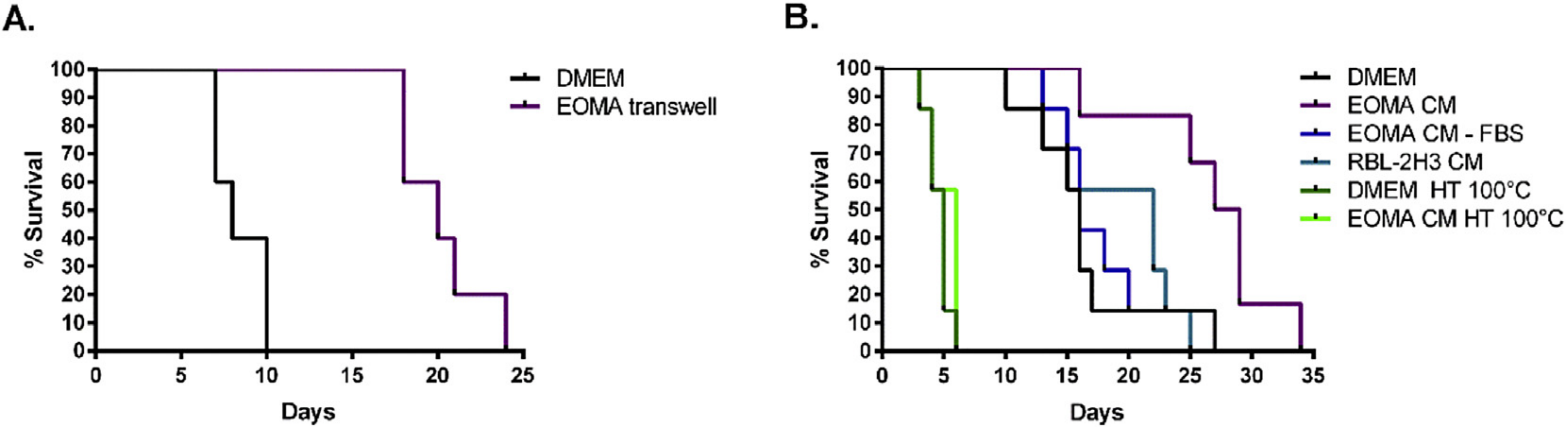
EOMA CM prolongs survival of adult filarial worms. (A) Culture of *L. sigmodontis* adult female worms in 0.22 μm transwell plates with EOMA cells with no media exchanged. Graph depicts a representative experiment. (B) *L. sigmodontis* adult female worms cultured in CM. One adult female worm was cultured per 1 ml of CM, and CM was exchanged every other day. EOMA CM significantly enhanced survival compared to DMEM (*p* = 0.012).

To determine whether the factors that prolong MF survival could support other developmental stages, we cultured *L. sigmodontis* adult female worms with EOMA cells in transwell plates. As with MF, adult *L. sigmodontis* survived longer when maintained in co-culture with EOMA cells in transwell plates than when cultured with media alone. In the absence of media exchanges, 50% of adult *L. sigmodontis* worms survive for 20 days when in transwell culture with EOMA cells and for only 7 days when cultured with DMEM (Fig. 7A).

Utilizing every other day exchanges with CM prolonged worm survival even more. As seen in Fig. 7B, 50% survival rate of adult *L. sigmodontis* worms was 28 days in EOMA CM and only 17 days in non-conditioned media (DMEM) (Fig. 7B). Increased survival was lost when worms were cultured in conditioned media obtained from EOMA cells grown in the absence of FBS (EOMA CM − FBS). As with MF, RBL-2H3 CM did not increase survival beyond that of control media (DMEM). Finally, heat treatment of EOMA CM and DMEM at 100 °C dropped survival below DMEM control, indicating it had a toxic effect. Overall, *in vitro* survival of adult *L. sigmodontis* filarial worms was consistently optimal in EOMA CM. As this media was also best for MF, these results suggest that there are common host-produced factors required by both MF and adult stages of filarial worms.

## 4 Discussion

In this study, we have shown that mouse endothelial cells (EOMA) and mosquito cells (C6/36) support the *in vitro* survival of *L. sigmodontis* MF and that EOMA conditioned media can be used to maintain both MF and adult filarial worms in long-term *in vitro* culture. Although co-culturing cells with MF results in the longest survival time, culturing MF in transwell plates or EOMA CM provide comparable results, indicating the cells produce soluble factors required for worm survival. EOMA cells require FBS to produce these factors, and the factors are sensitive to heat treatment and lipid removal. Dialysis experiments show that factors between 0.1 and 1 kDa are responsible for roughly half of the extended survival. Importantly, EOMA CM also supports the survival of *L. sigmodontis* adult worms.

The results of this study provide a robust method to maintain filarial worms in long-term *in vitro* culture using conditioned media from EOMA cells. Additionally, this study provides a starting point for the identification of host-produced factors required for filarial survival. Identification of these factors will be the focus of future work and will lead to both important biological insights regarding these parasites and the potential to develop a defined media for *in vitro* culture.

Although the *B. malayi* genome has been sequenced (Ghedin et al., 2007), a large portion is comprised of unique or hypothetical proteins. KEGG pathway analysis reveals that filarial worms lack many proteins involved in important pathways, including heme, riboflavin, and purine biosynthesis (Desjardins et al., 2013). This presents two possibilities: 1) filarial worms have evolved unique enzymes for the synthesis of essential nutrients, or 2) the worms acquire necessary precursors or end products from the host. *Wolbachia*, intracellular bacteria that reside within certain species of filarial worms, have been hypothesized to contribute to worm survival because they utilize biosynthesis pathways to produce heme, riboflavin and purines. However, because *Loa loa* filariae lack both *Wolbachia* as well as the aforementioned pathways, the contribution of *Wolbachia*-derived nutrients is likely minimal, with worms primarily relying on the host for these factors (Desjardins et al., 2013).

Unsupplemented DMEM contains important salts, metals, amino acids, and vitamins (including riboflavin). Heat treatment of riboflavin at 100 °C for 1 h has been shown to reduce the quantity by 50% (Farrer and Macewan, 1954), and may account for the decreased survival observed with heat treated DMEM. FBS contains trace amounts of hemoglobin and therefore may be an important source of heme (Wagner et al., 2007). Denaturation of hemoglobin is reversible at low temperatures but irreversible at 100 °C (Michnik et al., 2005), which may further explain why heating DMEM to high temperatures is toxic to MF (Fig. 3B).

Although filarial worms lack purine biosynthesis pathways, there is evidence they can convert pyrimidines to purines (Desjardins et al., 2013). Our data demonstrate that the addition of exogenous nucleosides does not enhance survival (Fig. 6B). However, MF may respond better to exogenous purine precursors or the addition of a carrier protein, as EOMA CM was enriched for nucleotide binding proteins compared to RBL-2H3 CM (Table 2).

Helminths also lack pathways for *de novo* synthesis of cholesterol. *W. bancrofti* (Rao and Sritharan, 1999) and *S. mansoni* (Chiang and Caulfield, 1989) have LDL receptors, indicating that these parasites may acquire cholesterol and other sterols from the host. In fact, adult schistosome worms contain cholesterol while cercariae contain sterols derived from the intermediate snail host (Furlong and Caulfield, 1988). Helminth infections have also been shown to alter the lipid profiles of infected patients (Bansal et al., 2005), though whether this is related to worm survival or disease pathogenesis remains unclear.

FBS contains cholesterol as well as lipid carriers HDL, LDL, VLDL, and chylomicrons. Lipid carriers are stable when subjected to low temperatures (56 °C) and denature at high temperatures (100 °C) (Jayaraman et al., 2006, Lu et al., 2012), therefore they would not be affected by 56 °C heat inactivation of FBS. Because culturing MF in DMEM with 10% FBS and supplementing with extra cholesterol does not promote long-term survival, cholesterol may be an important nutrient but play a minimal role in determining MF longevity.

EOMA cells grown in the absence of FBS (Fig. 2C) and chemically defined FBS replacements (Fig. 6A) failed to support MF survival. Although EOMA cells may be using FBS to synthesize and release factors, there is a possibility that the cells may simply be altering components present in FBS into derivatives that are more useful for the worms. For example, EOMA cells express endothelial lipase which can act on VLDL and chylomicrons to release triglycerides. Therefore, instead of directly producing nutrients MF require, EOMA cells may catalyze the release of sequestered nutrients already present in FBS.

Dialysis experiments indicate that factors between 0.1 and 1 kDa in size are responsible for half of the prolonged survival observed with EOMA CM (Fig. 4). Potential factors in the 0.1–1 kDa size range include vitamins, nucleotides, amino acids, monosaccharides, fatty acids, cholesterol, porphyrins (including heme), and lipopeptides. These small molecules are the building blocks for macromolecules, and the direct uptake of these factors from the host may enable filarial worms to bypass the requirement for several biochemical pathways. The small size of the beneficial factors may be critical, since MF and L3 stage larvae lack a fully developed intestinal tract (Laurence and Simpson, 1971, Beckett and Boothroyd, 1970) and thus may have difficulties acquiring macromolecules. Prior studies have shown that MF, and indeed all life cycle stages, have the ability to absorb low molecular weight substances directly through the cuticle (Smyth, 1990, Laurence and Simpson, 1971, Howells et al., 1987).

Given that none of the fractions resulted in complete restoration of MF survival similar to EOMA CM (i.e. median survival of 19 days), it is possible that necessary factors are also present in the larger fractions, and that the larger factors function as carrier proteins. Because small molecules would not be retained by the larger cutoffs, empty carrier proteins would lose their ability to prolong MF survival because their cargo is absent. Due to the heat and LRA-sensitive nature of EOMA CM, it likely contains both protein and lipid components that are beneficial for MF survival.

In addition to being a nutrient, factors in EOMA CM may provide an environmental cue or signal that promotes survival. Nematodes have cuticular sensilla that allow the parasite sense its location during its complex life cycle (Ashton et al., 1999). Neurons present in the sensilla can receive chemosensory, mechanosensory, and thermosensory signals (Ashton et al., 1999).

We found that the addition of recombinant mouse C3 slightly prolongs MF survival (Fig. 5B). Since C3 is known to interact with MF (Carter et al., 2007), it is possible that the C3 present in EOMA CM provides a chemosensory signal to indicate MF are located in the mammalian host. Future studies would need to administer repeat doses of C3 to see if survival can be further prolonged, as well as determine whether C3a or C3b is the important component. There are no C3a or C3b receptors in the *B. malayi* genome that are homologous to human complement receptors. The identification of filaria-specific complement receptors may provide useful targets for vaccines or rational drug design.

An in-depth understanding of the nutrients and environmental cues filarial worms need to survive is an important step toward the rational development of novel anthelmintics. The Global Program to Eliminate Lymphatic Filariasis would benefit greatly from effective short-course macrofilaricides, yet drug discovery is hindered by our inability to culture adult filarial worms. We have demonstrated that culturing adult *L. sigmodontis* worms in EOMA CM extends *in vitro* survival (Fig. 7). By prolonging the survival of filarial worms, investigators can employ high throughput screening to test candidate pharmaceuticals.

Prolonging *in vitro* survival is also the first step toward the development of an *in vitro* life cycle, which would shed light on the biology of larval development and could potentially lead to the identification of novel drug targets. Furthermore, worm recovery from animal hosts is often low, limiting the types of experiments that can be performed due to the lack of worm antigen and excretory-secretory product. An *in vitro* life cycle would bypass this problem by allowing for the expansion of filarial worms *in vitro* and mass production of worm antigen with high consistency.

A comparison of the nutrients, growth factors, and lipids present in FBS versus FBS replacements may provide insight into which classes of molecules are essential for worm survival. Due to high variability of FBS among lots, the ultimate goal would be to extend the *in vitro* survival of filarial worms in serum-free culture media. Future studies will also employ the use of mass spectrometry to chemically define factors present in the 0.1–1 kDa fraction of EOMA CM but absent in RBL-2H3 CM. Supplementing DMEM with the identified factors, and potentially carrier proteins, would be the next step toward the development of a defined culture media designed to support filarial worm survival.

## Figures and Tables

**Table 1:** Mass spectrometry identification of 32 spots exhibiting >1.5 fold upregulation in EOMA CM compared to RBL-2H3 CM.

Spot number	Match confidence	Protein name [species]	Accession no.	Protein MW	Protein pI
1	High	Serum albumin	ALBU_MOUSE	68,648	5.8
11	Low	Alstrom syndrome protein 1 homolog	ALMS1_MOUSE	359,993	6.0
15	High	Serum albumin	ALBU_MOUSE	68,648	5.8
18	High	Voltage-dependent R-type calcium channel subunit alpha-1E	CAC1E_MOUSE	257,071	8.5
25	High	Nucleolar RNA helicase 2	DDX21_MOUSE	93,493	9.2
28	High	Serum albumin	ALBU_MOUSE	68,648	5.8
30	High	Complement C4 [*Bos taurus*]	gi|31563307	101,820	6.2
36	High	Complement C3	CO3_MOUSE	186,366	6.3
41	High	Keratin, type II cytoskeletal 6B	K2C6B_MOUSE	60,285	8.5
42	High	Ectodysplasin-A receptor-associated adapter protein	EDAD_MOUSE	23,738	5.0
43	High	Ectodysplasin-A receptor-associated adapter protein	EDAD_MOUSE	23,738	5.0
44	High	Apolipoprotein E	APOE_MOUSE	35,844	5.6
46	High	Collagen alpha-1(I) chain	CO1A1_MOUSE	137,948	5.7
47	High	Inter-alpha (globulin) inhibitor H4 (plasma Kallikrein-sensitive glycoprotein) [*Bos taurus*]	gi|59857769	101,446	6.3
48	High	Serum albumin	ALBU_MOUSE	68,648	5.8
49	High	Cathepsin L1	CATL1_MOUSE	37,523	6.4
52	Low	Transcription factor EC	TFEC_MOUSE	35,122	6.3
53	Low	Very long-chain specific acyl-CoA dehydrogenase, mitochondrial	ACADV_MOUSE	70,831	8.9
54	High	Serum albumin	ALBU_MOUSE	68,648	5.8
55	High	Complement C3	CO3_MOUSE	186,366	6.3
56	High	Complement C3	CO3_MOUSE	186,366	6.3
58	High	Serum albumin	ALBU_MOUSE	68,648	5.8
61	High	Proteasome subunit alpha type-7	PSA7_MOUSE	27,838	8.6
65	High	Lymphoid-restricted membrane protein	LRMP_MOUSE	59,551	5.1
66	High	Peroxiredoxin-1	PRDX1_MOUSE	22,162	8.3
67	High	Centrosomal protein C10orf90 homolog	CJ090_MOUSE	71,476	8.8
68	High	C2 calcium-dependent domain-containing protein 4D	C2C4D_MOUSE	36,842	11.0
69	Low	Tripartite motif-containing protein 43B	TR43B_MOUSE	52,204	8.6
70	High	Nucleoside diphosphate kinase B	NDKB_MOUSE	17,352	7.0
78	High	Histone H2A.Z	H2AZ_MOUSE	13,545	10.6
79	High	E3 ubiquitin-protein ligase RNF181	RN181_MOUSE	19,088	5.7
80	Low	Replication factor C subunit 5	RFC5_MOUSE	38,072	7.7

All proteins were of murine origin, except for spot 30 (C4) and spot 47 (inter-alpha globulin inhibitor H4) which originated from *Bos taurus*. MW = molecular weight. pI = isoelectric point.

**Table 2:** Cluster analysis of proteins upregulated >1.5 fold in EOMA CM compared to RBL-2H3 CM.

Functional group	Genes	Accession no.
Regulation of Cell Death	Peroxiredoxin-1	PRDX1
	Nucleoside diphosphate kinase B	NDKB
Secreted/Extracellular	Albumin	ALBU
	Apolipoprotein E	APOE
	Complement C3	CO3
	Collagen alpha-1 chain	CO1A1
Nucleotide Binding	Nucleolar RNA helicase 2	DDX21
	Very long-chain specific acyl-CoA dehydrogenase (mitochondrial)	ACADV
	Nucleoside diphosphate kinase B	NDKB
	Replication factor C subunit 5	RFC5
Metal Ion Binding	Albumin	ALBU
	Voltage-dependent R-type calcium channel subunit alpha-1E	CAC1E
	Nucleoside diphospate kinase B	NDKB
	Replication factor C subunit 5	RFC5
